# Prevention of seminal vesicle damage by *Mucuna pruriens* var. *pruriens* seed extract in chronic unpredictable mild stress mice

**DOI:** 10.1080/13880209.2022.2157018

**Published:** 2022-12-23

**Authors:** Sitthichai Iamsaard, Somboon Kietinun, Jintana Sattayasai, Kingkan Bunluepuech, Alexander Tsang-Hsien Wu, Pannawat Choowong-In

**Affiliations:** a Department of Anatomy, Faculty of Medicine, Khon Kaen University, Khon Kaen, Thailand; b Research Institute for Human High Performance and Health Promotion (HHP & HP), Khon Kaen University, Khon Kaen, Thailand; c Department of Integrative Medicine, Chulabhorn International College of Medicine, Thammasat University, Pathum Thani, Thailand; d Department of Pharmacology, Faculty of Medicine, Khon Kaen University, Khon Kaen, Thailand; e Department of Applied Thai Traditional Medicine, School of Medicine, Walailak University, Nakhon Si Thammarat, Thailand; f TMU Research Center of Cancer Translational Medicine, Taipei Medical University, Taipei, Taiwan; g The PhD Program of Translational Medicine, College of Science and Technology, Taipei Medical University, Taipei, Taiwan; h Clinical Research Center, Taipei Medical University Hospital, Taipei Medical University, Taipei, Taiwan; i Graduate Institute of Medical Sciences, National Defense Medical Center, Taipei, Taiwan; j Center of Excellence in Marijuana, Hemp, and Kratom, Walailak University, Nakhon Si Thammarat, Thailand

**Keywords:** Seminal plasma, CUMS, apoptosis, Nrf2, TyrPho

## Abstract

**Context:**

Thai *Mucuna pruriens* (L.) DC. var. *pruriens* (Fabaceae) or T-MP seed extract has been shown to improve sexual performance and sperm quality.

**Objective:**

This study investigates the preventive effects of T-MP against seminal vesicle damage, apoptotic and Nrf2 protein expression in mice under chronic unpredictable mild stress (CUMS).

**Materials and methods:**

Forty-eight male ICR mice were divided into four groups: control, CUMS, T-MP300 + CUMS and T-MP600 + CUMS. Mice in control and CUMS groups received distilled water, while those in treated groups were pretreated with T-MP extract (300 or 600 mg/kg BW) for 14 consecutive days. The CMUS and co-treated groups were exposed to one random stressor (of 12 total) each day for 43 days. Components and histopathology of the seminal vesicle were examined, along with localization of androgen receptor (AR) and caspase 3. Expression of seminal AR, tyrosine phosphorylated (TyrPho), heat shock protein 70 (Hsp70), caspases (3 and 9) and nuclear factor erythroid 2-related factor 2 (Nrf2) proteins was investigated.

**Results:**

T-MP extract at a dose of 600 mg/kg BW improved seminal epithelial damage and secretion of fluid containing essential substances and proteins in CUMS mice. It also increased the expression of AR and TyrPho proteins. Additionally, T-MP increased expression of Nrf2 and inhibited seminal vesicular apoptosis through the suppression of Hsp70 and caspase expression.

**Conclusion:**

T-MP seeds have an antiapoptotic property in chronic stress seminal vesicle. It is possible to apply this extract for the enhancement of seminal plasma quality.

## Introduction

Chronic stress can cause various mental disorders, metabolic syndromes and immune system disorders and may affect the male reproductive system (Marin et al. 2011; Bergmann et al. 2014; Ilacqua et al. 2018; Zhang et al. 2020). Clinical studies have shown that psychological stress significantly increases serum cortisol levels and decreases sperm parameters, androgen levels and sexual performance (Shukla et al. 2010; Byun et al. 2013; Bhongade et al. 2015). The chronic unpredictable mild stress (CUMS) model is commonly used to study the effects of stress (Antoniuk et al. 2019). A previous study found that CUMS disrupts the integrity of the blood-testis barrier by decreasing the expression of zonula occludens-1 and claudin-11 (Kolbasi et al. 2021). Additionally, it can increase testicular heat-shock protein 70 (Hsp70), Bcl-2-associated X protein (Bax) and caspases 9 and 3, causing CUMS-induced cell apoptosis (Zou et al. 2019; Choowong-In et al. 2021b). In addition, CUMS has recently been found to damage the epididymis, impair sperm parameters and inhibit steroidogenic acute regulatory protein (StAR) and cytochrome P450 side chain cleavage (CYP450scc) enzyme expression in the testes (Fahim et al. 2019; Choowong-In et al. 2021a). Moreover, decreased expression of A-kinase anchoring protein 4 (AKAP4), androgen receptor (AR) and tyrosine phosphorylated (TyrPho) proteins are associated with reductions in sperm quality and testosterone (Choowong-In et al. 2021a).

Seminal vesicle (SV) is known to produce alkaline and viscous fluid containing fructose, proteins, potassium and phosphorus to facilitate the fertilizing ability of ejaculated sperm in the female reproductive tract (Drabovich et al. 2014). Changes of biochemical components in seminal fluid (SF) are involved in sperm capacitation, acrosomal exocytosis and sperm–zona interactions (Drabovich et al. 2014). The alteration of the SF components, especially fructosamine, was reported in infertile men and chronic stress animal models (Iamsaard et al. 2021). Additionally, the histologic changes of epithelial cells and decrease of androgen receptor (AR) expression in reproductive organs including seminal vesicle have been reported under chronic stress conditions (Arun et al. 2021; Iamsaard et al. 2021; Lapyuneyong et al. 2022). Interestingly, chronic stress can also induce apoptosis *via* increased Hsp70 and caspase 9 and 3 expression in the seminal vesicles, resulting in decreased epithelial height and seminal secretion (Iamsaard et al. 2020). Although antidepressant medications have been used to treat chronic stress, they have been shown to decrease sexual performance, semen parameters and fertility (Cartwright et al. 2016; Beeder and Samplaski 2020). Various studies have thus attempted to explore the potential of herbal medicines for counteracting the reproductive damage caused by chronic stress (Awodele et al. 2017; Nimrouzi et al. 2020; Dogani et al. 2022).

The seed of *Mucuna pruriens* (L.) DC. (Fabaceae) has traditionally been used as a source of herbal medicine in Ayurveda, often used in treating of neurodegenerative disorders, especially Parkinson disease, metabolic syndromes and male infertility (Divya et al. 2017). *M. pruriens* seeds are rich in various nutrient molecules, especially L-3,4-dihydroxyphenyl alanine (L-DOPA), and contain flavonoids and polyphenols (Misra and Wagner 2007; Longhi et al. 2011; Pathania et al. 2020). In addition, the seed extract possesses antioxidative, immunomodulation, antimicrobial, antiprotozoal, antifungal, analgesic, anti-inflammatory, anti-Parkinson’s, anti-depressant, antidiabetic, anticholesterolemic, antivenom and anticancer effects (Divya et al. 2017; Rai et al. 2017; Sinha et al. 2018; Pathania et al. 2020). Pharmacological studies showed that *M. pruriens* demonstrated repro-protective property by decreasing reactive oxygen species (ROS) production and increasing of antioxidant enzyme levels in sperm aged rat (Suresh et al. 2010). In previous studies, it was reported that *M. pruriens* can improve sexual behavior and libido induced in diabetic male rat (Suresh and Prakash 2012). Seed extract of *M. pruriens* can improve sperm parameters and increase serum sex hormone and catecholamine levels in infertile men (Shukla et al. 2009). Moreover, *M. pruriens* seeds can increase neuronal nitric oxide synthase (nNOS) and AR expression in the dorsal nerve of the penis (Seppan et al. 2020). In Thai folk medicine, Thai *M. pruriens* or T-MP has also been used to treat wounds, Parkinson’s disease, insect poisoning and male sexual dysfunction (Ayuraved Wittayarai Foundation 1998). T-MP seed extract has been prescribed for treatment of erectile dysfunction at a dosage of 1–2 g per 200 mL of warm water. Folk healers have also used T-MP seeds as a health tonic, aphrodisiac, and for male rejuvenation. Due to its antioxidant capacity, T-MP has been reported to enhance testosterone levels and sperm characteristics and to increase testicular AR, AKAP4 and TyrPho protein expression (Iamsaard et al. 2020). T-MP is quantified for L-DOPA and has been shown to improve sexual performance and testicular markers in CUMS mice (Choowong-In et al. 2021a). Indeed, T-MP extract has recently shown to protect only testicular injury and low sperm quality in stress and alcoholic animal models (Tangsrisakda et al. 2022; Choowong-In et al. 2021a). However, such effects have never been reported in the seminal vesicles of stress mice. This study thus aimed to investigate the protective effect of T-MP seed extract on seminal tissue and fluid impairment in CUMS mice.

## Materials and methods

### Plant collection and extraction

The aqueous T-MP seed extract used in this study was prepared as previously described (Iamsaard et al. 2020). Briefly, mature T-MP seeds were collected from the ripe pods of trees planted in Surin province, Thailand (14.8829°N, 103.4937°E). All plant samples were authenticated by Prof. Dr. Pranom Chantarnothai before collection as a botanical specimen voucher (code: S. Iamsaard 01) from the Khon Kaen University Herbarium (Faculty of Science). The seeds were washed with distilled water (DW) before sun drying. The dried seeds were crushed to form a coarse powder and then extracted with DW (ratio, 1:3 volume) at 80 °C for 30 min. Then, the aqueous seed extract solution was filtered using 0.22 mm filters and a lyophilized under spray dryer to obtain the T-MP seed powder (Faculty of Pharmaceutical Sciences, Khon Kaen University). The yield of the extract was approximately 16.29%. The extract fraction has been proven to contain the levodopa (L-DOPA) determined by nuclear magnetic resonance (NMR) spectrometry and high-performance liquid chromatography (HPLC) analyses.

### Animals and experimental design

ICR male mice (weighing 35–40 g, *n* = 48) were obtained from the Khon Kaen University Faculty of Medicine Animal Unit (Thailand) and housed in an animal laboratory (25 ± 2 °C, humidity 40–60%, and 12 h light/dark cycles) with access to food pellets and DW *ad libitum*. The experimental protocol was approved by the Khon Kaen University Animal Ethics Research Committee based on the guidelines laid out in the Ethics of Animal Experimentation of the National Research Council of Thailand (Rec. No. AEKKU 18/65). All animals were randomly divided into four groups (12 mice/group): (1) control (group I), (2) CUMS (group II), (3) T-MP300 + CUMS (group III) and (4) T-MP600 + CUMS groups (group IV). In groups I and II, mice were given distilled water (DW), whereas in groups III and IV, animals were pretreated by gastric feeding with T-MP seed extract (300 and 600 mg/kg BW, diluted with DW) for 14 consecutive days (pre-treatment period before stress induction) based on the method described by Choowong-In et al. (2021b). These selected doses have already proven to have no toxicity in rodents (Iamsaard et al. 2020; Choowong-In et al. 2021a). During the co-treatment period (CUMS period), mice in groups I and II were given DW, while those in groups III and IV were given seed extract from days 15 to 57 (43 days) based on the cycles of mouse spermatogenesis and sperm transit into the epididymis (Ray et al. 2014). After 1 h of treatment, mice in groups II–IV were subjected to one of the following unpredictable stressors each day chosen at random: (1) flashing light (6 h), (2) reversed light-dark cycle (12 h), (3) wet bedding (6 h), (4) cold-water forced swimming (5 min), (5) 45° cage tilting (6 h), (6) immobilization stress (6 h), 7) tail clamping (1 min), (8) water deprivation (6 h), (9) social isolation (6 h), (10) noise (6 h), (11) food deprivation (6 h), or (12) four rounds of electric foot shock (3 sec; Choowong-In et al. 2021a). At the end of the experiment, all mice were anesthetized with thiopental sodium (60 mg/kg BW, intraperitoneal injection, Jagsonpal Pharmaceuticals Ltd., India) and euthanized by cervical dislocation resulting in extensive damage to the brainstem and instantaneous unconsciousness before collection of the seminal vesicle.

### Collection of the seminal vesicle

Both seminal vesicles were dissected after scarification, and their relative weights were calculated using the following formula: relative weight (g/kg BW) = absolute organ weight (g) × 100 (g)/body weight (g) of animal (Aniagu et al. 2005). The right seminal vesicle of each animal was subsequently fixed in 10% neutral buffered formalin (48 h) before processing for routine histological investigation by hematoxylin and eosin (H&E) staining. The paraffin sections of the seminal vesicles were used for immunofluorescence staining against androgen receptor (AR) and caspase 3. Furthermore, the left seminal vesicle was snap-frozen in liquid nitrogen before studying the expression of specific proteins using western blotting.

### Biochemical component analysis of seminal fluid

For seminal fluid preparation, fresh seminal fluid was aspirated from the gland using a micropipette tip and transferred to an Eppendorf tube. The individual seminal fluid samples (*n* = 8/group) were homogenized with cold lysis buffer containing 1X radioimmunoprecipitation assay (RIPA) buffer (Cell signaling Technology, Inc., USA) and protease inhibitor cocktails (Sigma-Aldrich, Inc., USA) using glass grinder. After incubation for 30 min, the seminal extract was sonicated using an ultrasonicator (3 s, 20 times, 50 W) on ice and centrifuged at 12,000×*g* for 10 min at 4 °C to separate the seminal supernatant fluid from the pellet (Tongpan et al. 2019). The biochemical parameter levels of the seminal fluid were evaluated by radioimmunoassay at Srinagarind Hospital’s Clinical Laboratory Section (Khon Kaen University, Khon Kaen, Thailand).

### Histological observation

The fixed seminal samples were dehydrated and infiltrated with liquid paraffin (58–60 °C) for paraffin sectioning. All paraffinized-tissue blocks were sectioned at 5–7 µm thickness using a semi-automatic rotary microtome (ERM 3100, Hestion, Australia). The tissue sections were floated on warm water mixed with 0.5% gelatin at 45 °C before being mounted onto gelatin-coated slides. All sections were deparaffinized, rehydrated and stained by hematoxylin and eosin (Bio-optica, Italy) before mounting with dibutylphthalate polystyrene xylene (BDH Laboratory, UK). Finally, the histological features were observed under a light microscope (Nikon Light ECLIPSE E200, Japan), and the microphotographs were captured using a Nikon DXM1200 digital camera (Awodele et al. 2017).

### Western blotting analysis

For total protein extraction, the seminal vesicle tissue (50 mg) or seminal fluid (20 mg) from each animal was extracted with 1X radioimmunoprecipitation assay (RIPA) buffer (Cell Signaling Technology Inc., USA) containing protease inhibitor cocktails (Sigma-Aldrich, Inc., USA). The extract sample was homogenized using an ultrasonic probe (Cole-Parmer Instrument Company, Thailand). The total protein concentrations of each supernatant were measured three times using a NanoDrop ND-1000 spectrophotometer (Nanodrop Technologies, Wilmington, USA). The total proteins (300 µg) of each sample were mixed with loading buffer and boiled (95 °C, 5 min) before separation on 10% sodium dodecyl sulphate-polyacrylamide gel electrophoresis (SDS-PAGE). The separated proteins were transferred onto a nitrocellulose membrane (BIO-RAD, Hercules, CA, USA) at 200 volts for 50 min. The protein membranes were blocked with 5% bovine serum albumin (BSA) diluted in Tris-buffered saline containing 20% Tween 20 (TBST) for 1 h. Subsequently, each membrane was individually probed with primary antibody including mouse anti-AR, mouse monoclonal anti-Hsp70 (1:1000 dilution; Abcam, Cambridge UK), anti-phosphotyrosine, clone 4G10®, anti-caspase 9, anti-caspase3, anti-Nrf2 (1:1000 dilution; Merck Millipore corporation, Billerica, USA) diluted in 5% BSA in TBST, and mouse anti-glyceraldehyde 3-phosphate dehydrogenase (GAPDH, 1:10,000 dilution; Santa Cruz Biotechnology, Inc., USA) overnight at 4 °C diluted in 5% skim milk in TBST. After washing, each membrane was incubated with a 1:5000 dilution of anti-mouse IgG antibody conjugated with horseradish peroxidase (HRP; Santa Cruz Biotechnology, Inc., USA) for 1 h. Epidermal growth factor (EGF) lysate and bovine serum albumin (BSA) were used as positive and negative controls for TyrPho protein detection. Glyceraldehyde 3-phosphate dehydrogenase (GAPDH) was used as an internal control to confirm equal protein loading. Enhanced chemiluminescence (ECL) detection reagents (GE Healthcare Life Science, USA) were used as a commercial substrate before visualizing targeted proteins under gel doct 4 (ImageQuant 400, GH Healthcare, USA).

### Immunofluorescence staining

The paraffinized sections of seminal vesicle were deparaffinized and rehydrated with descending serial alcohols. The antigens on each section were retrieved by soaking in citrate buffer (10 mM citric acid, pH 6.0) and microwave heating at 560 Watts. After cooling, the boundary of the tissue section was circled on a glass slide using a peroxidase-antiperoxidase (PAP) pen (Millipore Co., USA). Then, endogenous peroxidase activity was inhibited with 3% hydrogen peroxide (H_2_O_2_) for 30 min and permeabilized in PBS containing 0.2% Triton X-100 for 10 min. All tissue sections were incubated with 3% BSA (Millipore Co., USA) in PBS for 1 h to block non-specific binding proteins. Then, the sections were probed with mouse monoclonal anti-androgen receptor (AR) or caspase 3 (1:200 dilution; Abcam, Cambridge, UK), whereas the primary antibody in the negative control section was omitted by incubation with PBS. After washing the unbound antibody, each section was further incubated with goat anti-mouse IgG (H + L) cross-adsorbed secondary antibody and Alexa Fluor 488 (1:300 dilution; Invitrogen™, USA) for 1 h. Then, tissue sections were incubated with Hoechst 33342 in PBS (1:10,000 dilution; Abcam, Cambridge, UK) for 10 min in a dark moisture chamber. After that, slides were washed and mounted with glycerol to be observed for immunofluorescence reactivity patterns under a fluorescence microscope using a fluorescein isothiocyanate (FITC) filter and photographed (Nikon ECLIPSE 80i) as previously described (Choowong-In et al. 2021b).

### Statistical analysis

All data were expressed as mean ± standard error of the mean (SEM) and analyzed using one-way analysis of variance (ANOVA) and followed by a Tukey *post hoc* test for multiple comparisons. A *P* value < 0.05 was considered to indicate a significant difference. The statistical analyses were performed using the SPSS statistics 19.0 (Statistical package for the social science, version 19.0, SPSS Inc., Armonk, New York, USA), downloaded from the Khon Kaen University Software.

## Results

### Protective effect of T-MP seed extract on CUMS seminal vesicles

**Figure 1.**
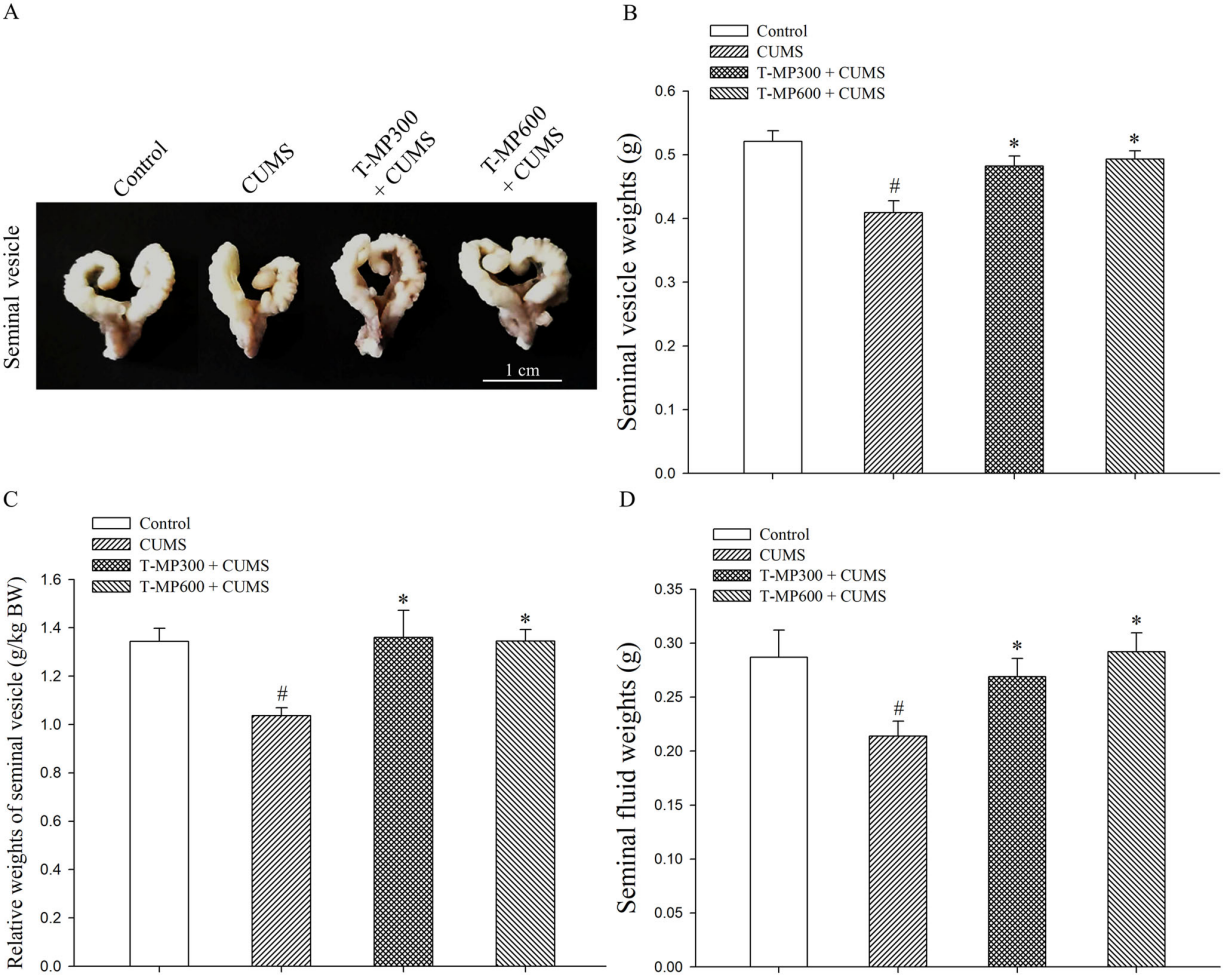
Representative photographs showing the (A) gross morphology, (B) absolute and (C) relative weights of the mouse seminal vesicles, and (D) fluid as compared among control, CUMS, and co-treated groups after co-treatment for 57 consecutive days (*n* = 12, each group). ^#^*p* < 0.05, statistically significant difference as compared between the control and CUMS groups. *p < 0.05, statistically significant difference as compared between the CUMS and co-treated groups.

Seminal vesicle (Figure 1B, C) and fluid (Figure 1D) weight were significantly lower in CUMS mice than in normal animals (SV weights, *F* = 4.34, *p* = 0.002; SV relative weights, *F* = 3.66. *p* = 0.001; SF weights, *F* = 3.35, *p* = 0.006). Administration with T-MP seed extracts at doses of 300 and 600 mg/kg BW significantly improved seminal vesicle parameters in the CUMS groups as compared to controls (Figure 1B–D; SV weights, *F* = 3.06, *p* = 0.02 and *F* = 3.77, *p* = 0.002; SV relative weights, *F* = 3.44, *p* = 0.0017 and *F* = 3.63, *p* = 0.001; SF weights, *F* = 3.67, *p* = 0.04 and *F* = 3.27, *p* = 0.003, respectively) consistent with their gross morphology, as demonstrated in Figure 1A.

### T-MP seed extract protected against CUMS-induced seminal epithelial injury

**Figure 2.**
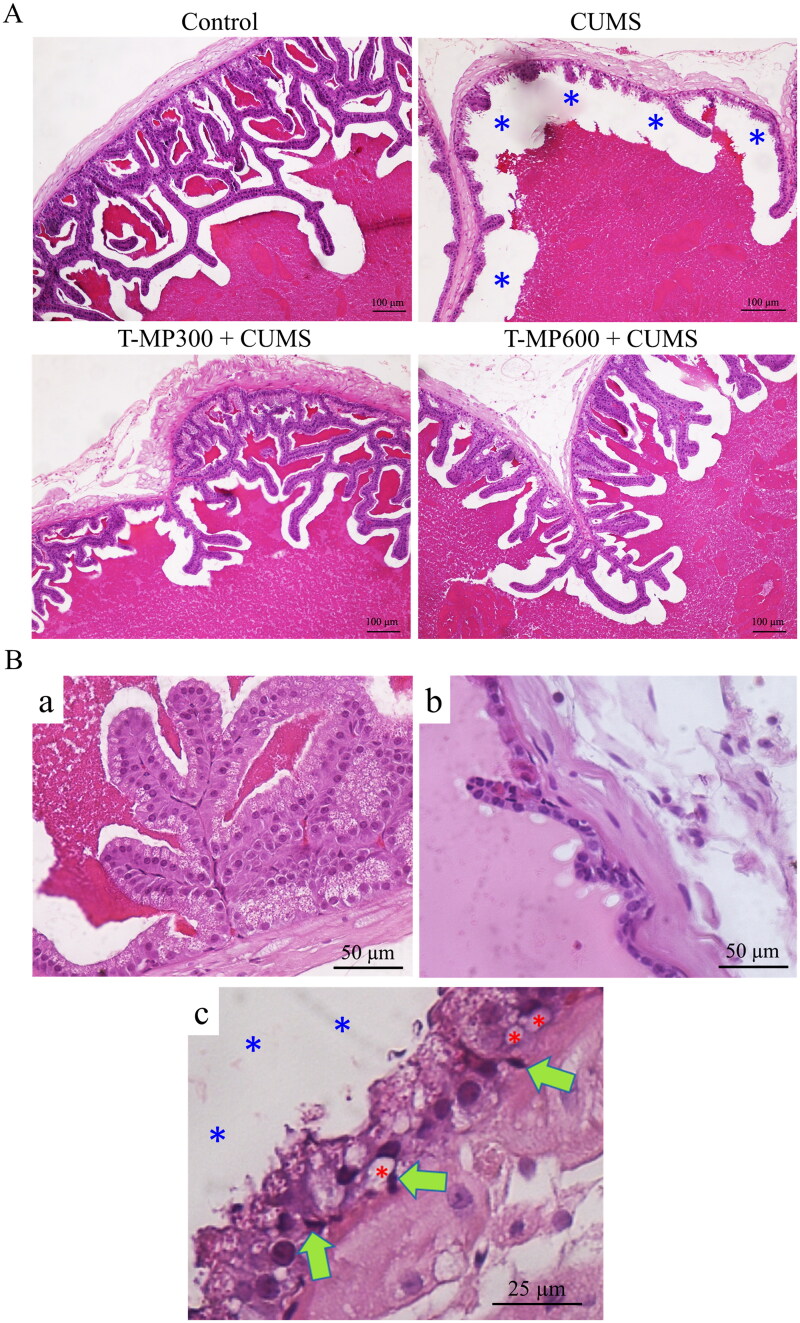
Representative microphotographs showing (A) histology of the seminal tissue and fluid strained by H&E, compared among control, CUMS and co-treated groups (T-MP 300 and 600 mg/kg BW). (Ba) normal seminal epithelium and histopathological features found in seminal vesicle of CUMS groups. (Bb) Decreasing of glandular epithelial cells. (Bc) Green arrows; pyknotic nuclei of basal cells, red asterisks; vacuolization within basal cells, blue asterisks; reduction of luminal seminal fluid.

The epithelial cells of the seminal vesicles in the control group were properly organized, whereas some seminal epithelial atrophy was observed in the CUMS group (Figure 2). Histopathological examination revealed vacuolization and pyknotic nuclei within basal cells, reductions in glandular epithelial cells, and reduction of luminal seminal fluid in the CUMS seminal epithelium as compared to controls.

### T-MP seed extract improved essential protein expression in CUMS seminal tissue

**Figure 3.**
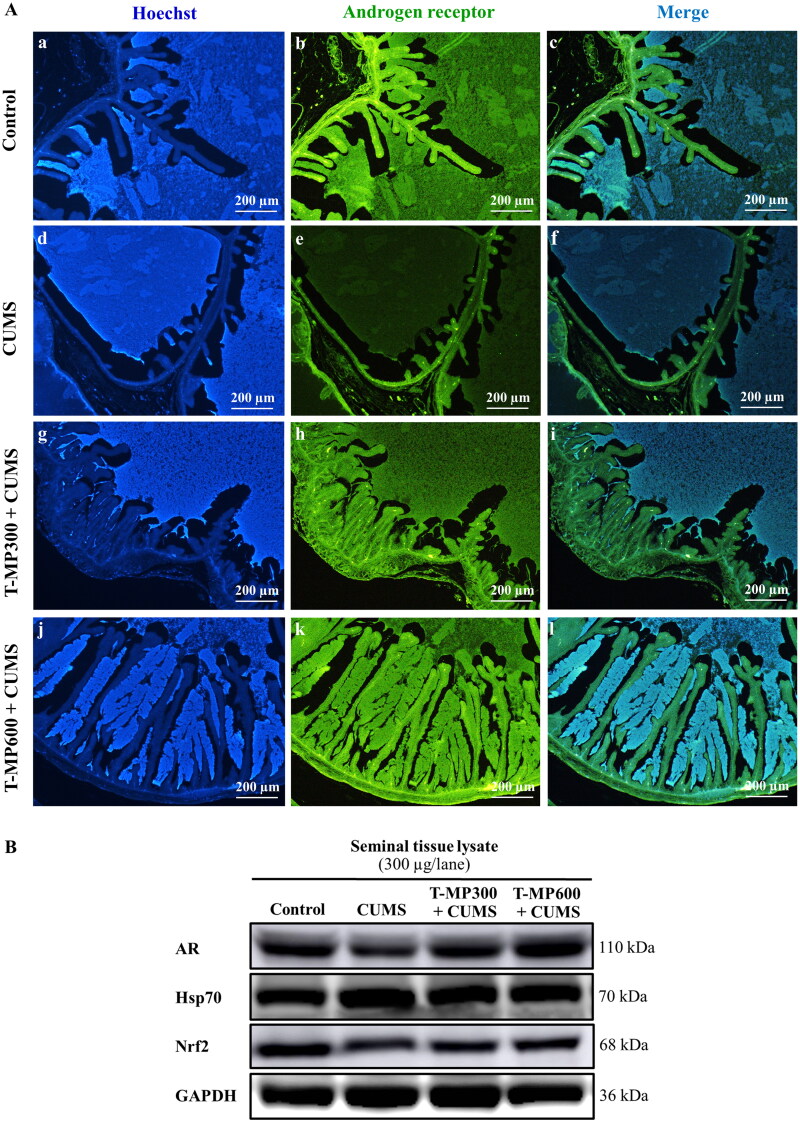
Immunofluorescence staining of the seminal vesicles compared among (Ab–c) control, (Ae–f) CUMS, (Ah–i) T-MP300 + CUMS, and (Ak–l) T-MP600 + CUMS groups against anti-androgen receptor (AR). Hoechst 33342 emitting blue fluorescence used as nuclear counterstain. AR immunostaining (green fluorescence). Expressions of (B) AR, heat shock protein 70 (Hsp70), and nuclear factor erythroid 2-related factor 2 (Nrf2) in seminal tissue compared among groups (*n* = 8, each group).

In the CUMS group, immunofluorescence staining for the androgen receptor (AR) showed low intensity in the seminal vesicle epithelium, luminal fluid and smooth muscle layer compared to the control group (Figure 3Aa–f). Interestingly, AR intensity was higher in CUMS seminal vesicles treated with T-MP seed extract compared to the untreated CUMS vesicles (Figure 3Ah-I, Ak-l). Western blotting confirmed that AR expression had improved remarkably in mice treated with T-MP at all doses (Figure 3B). Additionally, nuclear factor erythroid 2-related factor 2 (Nrf2) expression in the seminal vesicles was lower and that of heat shock protein 70 (Hsp70) was higher in CUMS compared to control mice (Figure 3B), both of which improved in the co-treated groups (Figure 3B).

### T-MP seed extract improved TyrPho protein expression in CUMS seminal tissue and plasma

**Figure 4.**
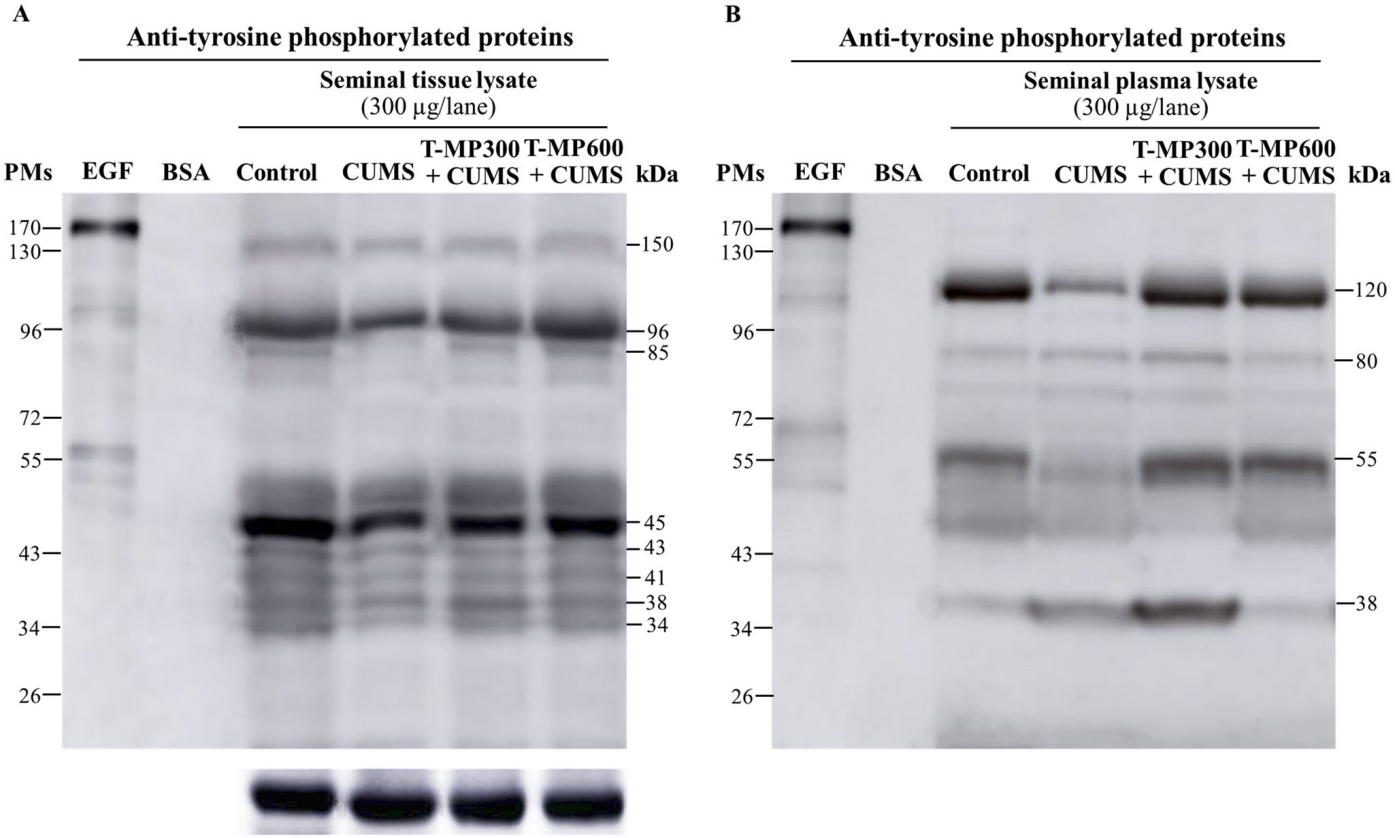
Expression of tyrosine phosphorylated (TyrPho) proteins observed in seminal tissue (A) and plasma lysates (B) compared among groups (*n* = 8, each group). EGF, epidermal growth factor used as positive control; BSA, bovine serum albumin used as negative control.

Decreased TyrPho protein expression was observed in seminal tissue (96, 85 and 45 kDa) and seminal plasma (120 and 55 kDa except 38 kDa) of CUMS mice compared to controls, but this decrease was ameliorated in the co-treatment groups (Figure 4A,B).

### T-MP seed extract suppressed caspases 3 and 9 expressions in CUMS seminal vesicles

**Figure 5.**
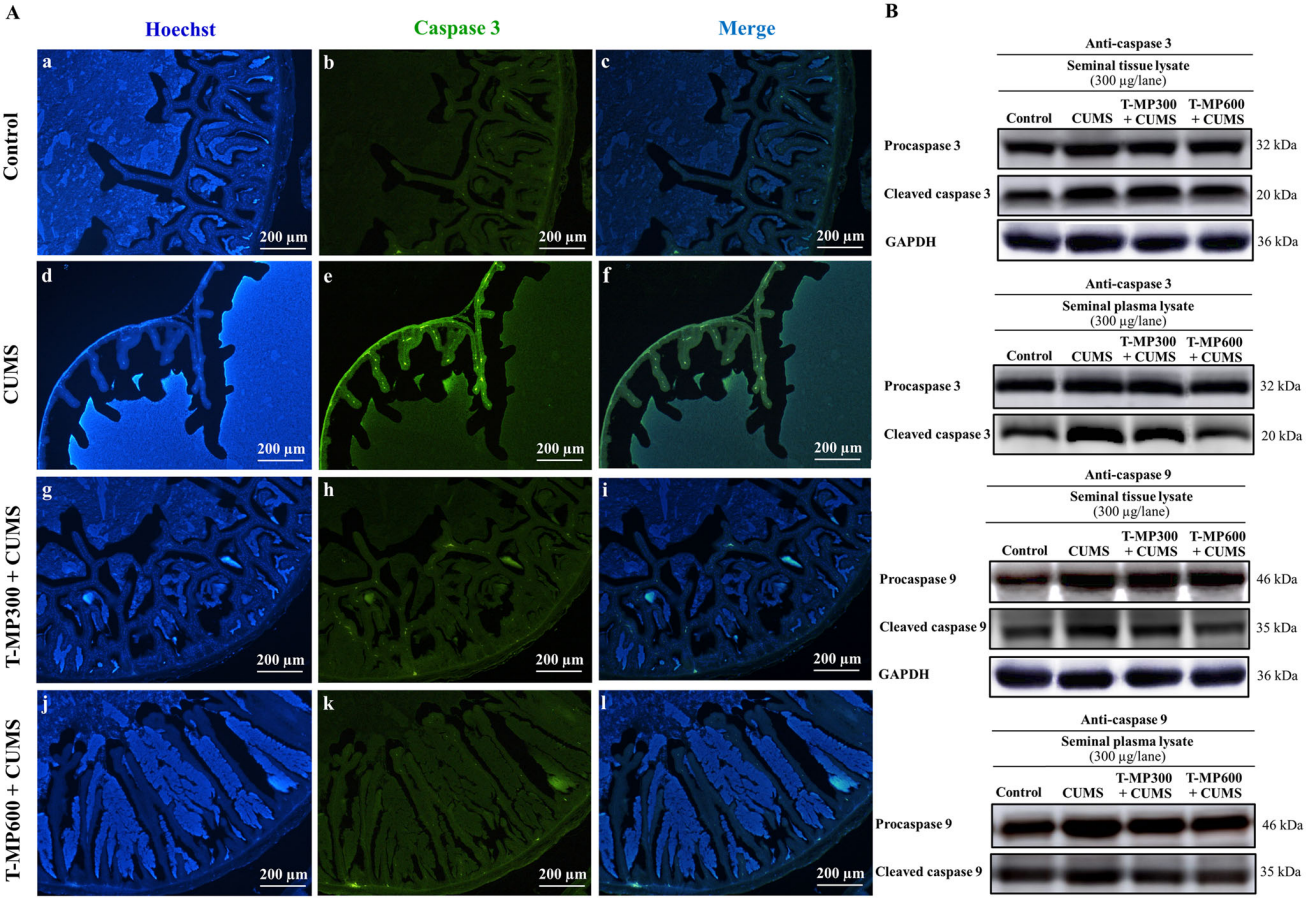
Immunofluorescence staining of seminal vesicle against anti-caspase 3 (A). Nuclei stained by Hoechst 33342 (blue-emitting fluorescent). Expression of pro and cleaved caspases 3 and 9 in rat seminal tissue and plasma lysates compared among control, CUMS and treated groups (B) (*n* = 8, each group).

The intensity of caspase 3 in the CUMS seminal vesicles was higher, indicating some apoptosis (Figure 5Ae-f). After T-MP seed extract administration, the caspase 3 intensity was decreased, suggesting suppression of apoptosis (Figure 5Ah-I, Ak-l). Western blotting confirmed that the expression of caspases 3 and 9 with pro and cleaved forms was higher in CUMS seminal tissue and plasma (Figure 5B). However, T-MP seed extract at all doses (especially at 600 mg/kg BW) reduced caspase protein expression (Figure 5B).

### Effect of T-MP seed extract on changes in the biochemical components of seminal fluid

Changes in biochemical parameters of the seminal fluid of control, CUMS and co-treated mice are shown in Table 1. Significantly, seminal plasma parameters, including total protein, aspartate aminotransferase (AST, *F* = 8.19, *p* = 0.001), aminotransferase (ALT, *F* = 7.51, *p* = 0.007), chloride (*F* = 4.04, *p* = 0.03), calcium (*F* = 5.63, *p* = 0.05), magnesium (*F* = 5.47, *p* = 0.02), phosphorus (*F* = 5.61, *p* = 0.003), fructosamine (*F* = 9.33, *p* = 0.001) and prostate-specific antigen (PSA, *F* = 12.85, *p* = 0.002), were lower in CUMS mice compared to controls. These parameters improved after co-treatment with T-MP seed extract at the doses of 300 and 600 mg/kg BW (AST, *F* = 8.19, *p* = 0.001 and *F* = 8.56, *p* = 0.004; ALT, *F* = 8.08, *p* = 0.025 and *F* = 8.10, *p* = 0.03; chloride, *F* = 5.20, *p* = 0.003 and *F* = 5.62, *p* = 0.005; calcium, *F* = 4.75, *p* = 0.04 and *F* = 4.00, *p* = 0.019; magnesium, *F* = 5.60, *p* = 0.001 and *F* = 5.47, *p* = 0.002; phosphorus, *F* = 4.72, *p* = 0.001 and *F* = 6.12, *p* = 0.002; fructosamine, *F* = 11.00, *p* = 0.001 and *F* = 11.00, *p* = 0.001; PSA, *F* = 13.10, *p* = 0.002 and *F* = 13.83, *p* = 0.002, respectively). However, although seminal albumin, sodium, potassium and bicarbonate levels were higher in the co-treated groups than in the CUMS group, these differences were not statistically significant (Table 1).

**Table 1.** Comparison of biochemical component parameters in the seminal fluid of the control, CUMS and co-treated groups (*n* = 8, each group).

Biochemical parameters in seminal fluid	Groups			
	Control	CUMS	T-MP300 +CUMS	T-MP600 +CUMS
Total protein (g/dL)	21.21 ± 0.11	20.16 ± 0.09^#^	20.79 ± 0.09	21.88 ± 0.01*
Albumin (g/dL)	0.17 ± 0.03	0.13 ± 0.03	0.17 ± 0.03	0.17 ± 0.03
ALT (U/L)	10.00 ± 1.00	4.67 ± 0.33^#^	9.33 ± 1.67*	10.00 ± 1.53*
AST (U/L)	58.67 ± 0.88	21.33 ± 3.33^#^	55.67 ± 2.19*	57.33 ± 4.91*
Sodium (mEq/L)	173.33 ± 2.96	169.33 ± 2.73	171.33 ± 0.33	173.67 ± 1.76
Potassium (mEq/L)	1.47 ± 0.03	1.40 ± 0.06	1.43 ± 0.03	1.47 ± 0.03
Chloride (mEq/L)	139.00 ± 0.58	136.67 ± 0.33^#^	139.67 ± 0.33*	139.33 ± 0.33*
Bicarbonate (mEq/L)	1.67 ± 0.33	0.67 ± 0.17	1.50 ± 0.29	1.67 ± 0.33
Calcium (mg/dL)	2.40 ± 0.35	1.03 ± 0.03^#^	2.17 ± 0.33*	2.30 ± 0.15*
Magnesium (mg/dL)	0.24 ± 0.00	0.21 ± 0.00^#^	0.24 ± 0.00*	0.24 ± 0.00*
Phosphorus (mg/dL)	3.63 ± 0.22	2.17 ± 0.07^#^	3.07 ± 0.54*	3.77 ± 0.21*
Fructosamine (µmol/L)	65.67 ± 4.70	52.67 ± 1.20^#^	64.00 ± 0.58*	64.00 ± 0.58*
PSA (µmol/L)	0.03 ± 0.00	0.02 ± 0.00^#^	0.04 ± 0.00*	0.04 ± 0.00*

Data represented as mean ± standard error of the mean (SEM).

#*p* < 0.05, statistically significant difference as compared between the control and CUMS groups.

*p < 0.05, statistically significant difference as compared between the CUMS and co-treated groups.

## Discussion

T-MP seed has been traditionally used in treating dysuria and improving erectile dysfunction. It exhibited high antioxidant capacity and has been regarded as safe in acute toxicity in male rats (Iamsaard et al. 2020). Data from the current work also demonstrated that T-MP seed extract containing L-DOPA can improve sexual behaviors and reduce corticosterone to improve testosterone level resulting in decreased testicular apoptosis and increased spermatogenesis especially improvement of AKAP4 and TyrPho proteins (Choowong-In et al. 2021a, 2021b). This improvement also occurs in the epididymis resulted in increased sperm quality in chronic stress. However, the protective effect of T-MP seed extract in seminal vesicle of CUMS mice remain uncertain. In this study, we explored the protective effect of T-MP seed extract against CUMS-induced seminal vesicle damage in mice for the possibility of its increasing of androgen receptor and Nrf2 and suppression of apoptosis in seminal tissue. Such seminal vesicle atrophy was associated with decreased AR expression and resulted in decreased secretion of seminal fluid, which contains various essential nutrients and proteins. Insufficient secretion from the seminal vesicles can inhibit sperm maturation, motility, capacitation and acrosome reaction in the female reproductive tract (Juyena and Stelletta 2012). Moreover, CUMS induction may reduce the expression of AR in the smooth muscular layer of the seminal vesicles, affecting seminal fluid secretion into the lumen (Welsh et al. 2010). Our findings showed that CUMS increased caspases 3 and 9 expressions in the seminal vesicles, corresponding to damage to the seminal epithelium (Iamsaard et al. 2021). This was consistent with the increased intensity of caspase-3 in the seminal epithelium of CUMS mice, as revealed by immunofluorescence. Decreases in androgen levels have been shown to increase apoptosis in the seminal epithelium of castrated animals (Tanji et al. 2003). In histopathology, vacuolization and pyknotic nuclei within basal cells may be involved in cellular apoptosis *via* increasing of caspase 3 and 9 expressions. In addition, reductions in glandular epithelial cells may involve in decreasing of AR expression within seminal epithelium. It might affect synthesis and secretion of seminal exosomes resulting in reduction of luminal seminal fluid in the CUMS seminal epithelium that play a major role in sperm motility and maintaining survival capacity in the female reproductive tract (Vickram et al. 2021). Hsp70 can also suppress apoptosis by inhibiting procaspase 9 from forming of apoptotic protease activating factor 1 (Apaf-1) oligomers, known as apoptosome complex (Beere et al. 2000), which may block the release of cytochrome C from mitochondria by inhibiting Bcl-2-associated X (Bax) activation (Stankiewicz et al. 2005). We suggest that CUMS may damage the mitochondrial membrane *via* the procaspase 9 and 3 apoptotic signaling pathway. Additionally, excess cellular levels of reactive oxygen species (ROS) cause an unbalancing of the redox reaction, leading to increased apoptosis (Redza-Dutordoir and Averill-Bates 2016). Moreover, nuclear factor erythroid 2-related factor-2 (Nrf2) is an essential transcription factor for cell defense against ROS in controlling antioxidant gene expression (Saha et al. 2020). A recent study demonstrated that CUMS decreased Nrf2 expression in mouse seminal vesicles, suggesting that CUMS may increase the oxidative stress in the seminal vesicles *via* the Nrf2 pathway to decrease antioxidant enzyme production. T-MP seed extract has recently been found to contain levodopa (L-DOPA) and phenolic compounds and exhibit potent antioxidant capacity (Iamsaard et al. 2020; Choowong-In et al. 2021b). In addition, T-MP seeds are rich in nutrients such as amino acids (aspartic acid, glutamic acid, arginine, leucine, isoleucine and L-DOPA), fatty acids (palmitic, stearic, oleic and linoleic acids), vitamins (niacin and ascorbic acid), minerals (K^+^, Ca^2+^, P^3−^ and Mg^2+^), and phenolic compounds (quercetin and myricetin) (Misra and Wagner 2007; Ahmad et al. 2008; Pathania et al. 2020). Additionally, the morin hydrate and naringin contained in this related plant have been shown to demonstrate the antistress property (Elizabeth et al. 2020; Oladapo et al. 2021). Those bioactive compounds are potentially therapeutic agents for the treatment of chronic diseases such as Parkinson’s, diabetic mellitus and male infertility (Lampariello et al. 2012). Previous studies have reported that the seed extract of *M. pruriens* is able improve male infertility caused by chronic stress in both human and animal models (Mahajan et al. 2012; Lapyuneyong et al. 2022). In stress rats, T-MP seed extract not only improves male sexual performance but has also been shown to improve testicular markers involved in spermatogenesis and testosterone synthesis (Lapyuneyong et al. 2022; Choowong-In et al. 2021b). The ability of T-MP seed extract to alleviate seminal damage and apoptosis might be result of its high antioxidant capacities previously demonstrated in Iamsaard et al. (2020). Indeed, L-DOPA found in T-MP seeds has been shown to exhibit antioxidant, free radical scavenging and metal chelating activity (Cacciatore et al. 2018; Choowong-In et al. 2021b) and is involved in the deprotonation of hydroxyl groups to rapidly transfer electrons from phenolate anions to free radicals (Jodko et al. 2022).

The improved AR expression observed in the co-treated groups was consistent with the morphological features and functions of the seminal vesicles. Additionally, T-MP improved Nrf2 expression in the seminal vesicles of CUMS mice, suggesting recovery of antioxidant protein expression and protection against oxidative tissue damage. Furthermore, the increased testosterone levels in T-MP-treated CUMS mice might be involved in the mitigation of seminal damage (Choowong-In et al. 2021b). In this study, T-MP seed extract suppressed Hsp70, and caspases 9 and 3 expression in the seminal vesicles, suggesting it may protect against CUMS-induced mitochondrial membrane damage that occurs *via* the procaspases 9 and 3 apoptotic pathway. Moreover, the increased levels of trace elements (chloride, calcium, magnesium and phosphorus) in the seminal fluid of the T-MP treated groups were associated with sperm physiology, particularly that of fructosamine, which is a primary energy source for sperm and a major carbohydrate in seminal plasma. The increased fructosamine levels in the co-treated groups might facilitate sperm hyperactive motility and capacitation after ejaculation. Additionally, the increased prostate-specific antigen (PSA) levels observed in our study are assumed to be involved in the liquefaction and fertilization process. Moreover, the increased levels of seminal plasma enzymes (aspartate aminotransferase, AST; alanine aminotransferase, ALT) observed in the co-treated groups may relate to spermatic density and motility. This indicates that T-MP seed extract may improve seminal fluid quality in CUMS, thus improving male fertility.

Various proteins secreted from the male reproductive tract have been shown to play roles in sperm motility, capacitation and acrosome reaction (Naz and Rajesh 2004; Lin et al. 2006; Ickowicz et al. 2012). One of those essential proteins is tyrosine phosphorylated (TyrPho) protein, which is expressed in seminiferous epithelial cells, epididymal cells and seminal epithelial cells (Chaichun et al. 2017; Sawatpanich et al. 2018; Tongpan et al. 2019). Chronic stress-induced changes in TyrPho protein expression are involved in reproductive tissue damage, low sperm quality parameters and decreased testosterone levels (Arun et al. 2021; Lapyuneyong et al. 2022; Choowong-In et al. 2021a). Interestingly, the improved TyrPho protein expression (96, 85 and 45 kDa) in the seminal tissue and fluid of CUMS mice treated with T-MP might be a consequence of AR expression and testosterone action stimulating biosynthesis of seminal plasma proteins (Arun et al. 2016; Tongpan et al. 2019; Yannasithinon and Iamsaard 2019). Additionally, increases in 120 and 55 kDa TyrPho protein expression might facilitate secretory protein biogenesis before ejaculation (Drabovich et al. 2014). Although these findings are promising, the functions of some TyrPho proteins observed in CUMS mice require further elucidation. In addition, data on ROS levels and antioxidant enzyme activities are required to confirm the improvement of Nrf2 expression with T-MP coadministration. Quercetin is known to improve oxidative stress‑induced cell apoptosis of seminal vesicles *via* inhibiting Nrf2 in type 1 diabetic rats (Dong et al. 2022). In addition, heme oxygenase-1 (HO-1) is an essential cytoprotective enzyme as downstream of Nrf2 that can degrade heme to carbon monoxide, free iron and biliverdin, degrading to bilirubin (Loboda et al. 2016). The beneficial effects of HO-1 include protection against oxidative injury, regulation of apoptosis, modulation of inflammation as well as contribution to angiogenesis (Loboda et al. 2016). Such fundamental reasons are related to the Nrf2/HO1 pathway that may be involved in prevention of seminal vesicle damage by T-MP extract administration.

A limitation of this study is the lack of transcriptomic analysis of the seminal vesicle responses to the reproductive CUMS to confirm the associations and mechanisms among TyrPho proteins (Skerrett-Byrne et al. 2021). Nevertheless, some TyrPho proteins have been characterized for their functions, especially involved in exosome composition and seminal plasma proteome (Candenas and Chianese 2020). It is possible to further investigate the role of Nrf2/HO-1 in the mechanism of the protective effect of T-MP extract against CUMS-induced seminal vesicle damage.

## Conclusions

This study demonstrated the potential ability of T-MP aqueous seed extract (especially at a high dose) to improve seminal vesicle morphology and secretion including androgen receptor, Hsp70, caspases (3 and 9), Nrf2 and tyrosine phosphorylation expression.
